# Trial baseline characteristics of a cluster randomised controlled trial of a school-located obesity prevention programme; the Healthy Lifestyles Programme (HeLP) trial

**DOI:** 10.1186/s12889-017-4196-9

**Published:** 2017-04-04

**Authors:** Jenny Lloyd, Siobhan Creanor, Lisa Price, Charles Abraham, Sarah Dean, Colin Green, Melvyn Hillsdon, Virginia Pearson, Rod S. Taylor, Richard Tomlinson, Stuart Logan, Alison Hurst, Emma Ryan, Wendy Daurge, Katrina Wyatt

**Affiliations:** 1 University of Exeter Medical School, University of Exeter St Luke’s Campus, Heavitree Road, Exeter, EX1 2LU UK; 2 Peninsula Clinical Trials Unit and Biostatistics, Bioinformatics & Biomarkers Group, Plymouth University Peninsula Schools of Medicine & Dentistry (formerly Peninsula College of Medicine and Dentistry), ITTC Building, Plymouth Science Park, Plymouth, Devon PL6 8BX UK; 3 Sport and Health Sciences, College of Life and Environmental Sciences, University of Exeter St Luke’s Campus, Heavitree Road, Exeter, EX1 2LU UK; 4 Department of Child Health Barrack Road, Royal Devon and Exeter Hospital, Royal Devon & Exeter NHS Trust, Exeter, EX2 5DW UK; 5 Public Health Devon, Devon County Council, County Hall, Topsham Road, Exeter, EX2 4QL UK; 6 Isca Academy, Devon, EX2 6AP UK; 7 St Leonard’s Primary School, Exeter, Devon EX2 4NQ UK

**Keywords:** Cluster randomised controlled trial, Child, School, Obesity prevention, Complex intervention

## Abstract

**Background:**

We have developed a healthy lifestyles programme (HeLP) for primary school aged children (9–10 years), currently being evaluated in a definitive cluster randomised controlled trial. This paper descriptively presents the baseline characteristics of trial children (BMI, waist circumference, % body fat, diet and physical activity) by gender, cluster level socio-economic status, school size and time of recruitment into the trial.

**Methods:**

Schools were recruited from across the South West of England and allocated 1:1 to either intervention (HeLP) or control (usual practice) stratified by the proportion of children eligible for free school meals (FSM, <19%, ≥19%) and school size (one Year 5 class, >1 Year 5 class). The primary outcome is change in body mass index standard deviation score (BMI sds) at 24 months post-randomisation. Secondary outcomes are BMI sds at 18 months, waist circumference and percentage body fat sds at 18 and 24 months, proportion of children classified as underweight, overweight and obese at 18 and 24 months, physical activity (for a sub-sample) and food intake at 18 months.

**Results:**

At baseline 11.4% and 13.6% of children were categorised as overweight or obese respectively. A higher percentage of girls than boys (25.3% vs 24.8%) and children from schools in FSM category 2 (28.2% vs 23.2%) were overweight or obese. Children were consuming a mean (range) of 4.15 (0–13) energy dense snacks (EDS) and 3.23 (0–9) healthy snacks (HS) per day with children from schools in FSM category 2 consuming more EDS and negative food markers and less HS and positive food markers. Children spent an average 53.6 min per day (11.9 to 124.8) in MVPA and thirteen hours (779.3 min) per day (11 h to 15 h) doing less than ‘light’ intensity activity. Less than 5% of children achieved the Departments of Health’s recommendation of 60 min of MVPA every day.

**Conclusion:**

We have excellent completeness of baseline data for all measures and have achieved compliance to accelerometry not seen before in other large scale studies. Our anthropometric baseline data is representative of local and national data for children this age and reflects the gender and socio-economic variations expected of children this age in relation to physical activity and weight status.

**Trial registration:**

ISRCTN15811706 (1/05/2012).

## Background

Currently one fifth of children in the UK are overweight or obese when they begin school and this figure increases to one third by the time they leave primary school [1]. Furthermore, children from the most economically deprived neighbourhoods are twice as likely to be obese at both reception and Year 6 than those from the most affluent neighbourhoods [1]. Obesity is therefore not only a serious problem for individual children and the wider population; it is also a significant contributor to health inequality. Despite obesity being classed by the WHO as ‘one of the greatest public health challenges of the twenty-first century [2], there is limited evidence for programmes which can effectively engage children and their families from all socio-economic groups to alter their diet and physical activity behaviours.

The relative contribution of physical activity, sedentary activity and diet to the development of obesity in children is unclear, partly because these variables are difficult to accurately measure and the balance of energy is complex [3, 4]. In addition, these lifestyle factors also interact with genetic factors affecting people’s propensity to gain weight, thus creating a highly individualised complex equation of factors leading to the development of obesity. However, prolonged periods of sitting (e.g. TV viewing/screen-based activity) [5], low levels of physical activity [6], parents’ inactivity [7] and high consumption of dietary fat, carbohydrate and sweetened fizzy drinks [8–10] have all been identified as common and modifiable risk factors that can be targeted in school-based interventions.

It is unsurprising that most childhood obesity prevention programmes to date have been situated within the school environment, particularly when schools’ existing organisational, social and communication structures provide opportunities for regular health education and the possibility of a health promoting environment. In addition, schools have the potential to reach children and their families across the social spectrum; however, despite the increasing number of school-based interventions designed to prevent obesity in children, there remains limited evidence of effective interventions. Results from systematic reviews of school-based interventions have been inconsistent [11–13] and a 2012 review of reviews examining the evidence from systematic reviews of school-based programmes in the control and prevention of childhood obesity, concluded that, whilst there was weak to moderate evidence for diet and physical activity combined interventions, the effect sizes were small and an understanding of the necessary conditions that lead to the sustained behaviour change necessary to affect weight status remains elusive [14]. A recently reported trial of a school-based intervention aimed at increasing physical activity, reducing sedentary behaviour and increasing fruit and vegetable consumption in children aged 9–10 was found to be ineffective [15]. The authors concluded that future interventions need to be more intensive and embed diet and physical activity behaviour change across the curriculum. In addition, interventions should aim to increase knowledge, motivation and skills of the children involved, find ways to increase child engagement in programme content and, crucially, engage parents [16]. Based on the lessons learnt from previous school-based interventions, we developed the Healthy Lifestyles Programme; a school-based obesity prevention intervention.

The aim of the overall study is to assess the effectiveness and cost effectiveness of the Healthy Lifestyles Programme in a cluster randomised controlled trial. This paper, however, focuses on the baseline characteristics of all the trial children as a representative, large cohort of 9–10 year olds. We descriptively present and discuss their BMI, waist circumference, % body fat, diet and physical activity by gender, cluster level socio-economic status, school size and time of recruitment into the trial (2012 or 2013). We obtained both anthropometric and behavioural measures from 99% of the original cohort, making this dataset a representative sample of children this age and comparisons with the national data on weight and socioeconomic status show that it is representative of children in the UK.

### The Healthy Lifestyles Programme (HeLP)

The Healthy Lifestyles Programme is a multi-component school-based obesity prevention intervention which delivers a general healthy lifestyle message, encouraging a healthy energy balance with a focus on behaviours relating to the consumption of sweetened fizzy drinks; healthy and unhealthy snacks, physical activity and reducing screen time.

In order to develop the intervention we followed the Medical Research Council’s framework for the development and evaluation of complex interventions [17] and used an intervention mapping protocol [18]. Details of this process are reported elsewhere [19].

The programme takes a whole school approach and activities are delivered over four phases which have been ordered to enable and support behaviour change [20]. Early development and piloting began in 2006 and details of the conceptualisation of HeLP can be found elsewhere [20]. The results from the definitive cluster randomised controlled trial will be available in Spring 2017.

## Methods

We are undertaking a definitive cluster randomised controlled trial to assess the effectiveness and cost-effectiveness of HeLP in preventing overweight and obesity in children. The primary outcome is change in body mass index standard deviation score (BMI sds) at 24 months post-randomisation. Secondary outcomes are BMI sds at 18 months, waist circumference sds at 18 and 24 months, percentage body fat sds at 18 and 24 months, proportion of children classified as underweight, overweight and obese at 18 and 24 months, objectively measured physical activity at 18 months and self-reported food intake (weekday and weekend) at 18 months. The costs associated with the delivery of the HeLP intervention and its cost-effectiveness versus usual practice will be determined and a mixed-methods process evaluation is exploring the way the Programme works (that is, how it was delivered, taken up, and experienced). A questionnaire bespoke to the trial called the My Lifestyle Questionnaire (MLQ) has been designed to assess possible mediators of change. This questionnaire was distributed at baseline and 12 months post-baseline (i.e. immediately following the end of the intervention period) to all children in the trial. This paper focuses specifically on the baseline data of weight status, diet, physical activity and sedentary behaviour for all children and presents these data by gender, cluster level socio-economic status, school size and time of recruitment into the trial (2012 or 2013). We do not present the data by allocated group (intervention v control) as baseline data was collected prior to group allocation being revealed by the Clinical Trials Unit.

### Recruitment of schools and children

Ethical approval for the trial was obtained from the Peninsula College of Medicine and Dentistry in March 2012 (reference number 12/03/140). The recruitment target for this trial was 32 schools, to allow for a conservative 20% attrition rate by 24 months (196 children): 24 month data was required from 760 children to achieve 90% power in the primary analysis [21]. All schools were recruited by July 2012 and children were recruited prior to baseline measures (2012 for cohort 1 schools and 2013 for cohort 2 schools). Given all 32 schools recruited remained in the trial and the total number of children in Year 5 classes was higher than originally estimated, substantially more children were recruited than the target of 980 children.

Schools from across Devon were recruited via the Devon Association of Primary School Heads (DAPH) and local primary school learning community meetings. The inclusion criteria were state primary and junior schools with children in at least one single Year 5 group of 20 or more children. At the start of the trial we estimated that approximately 125 schools were eligible. All children in all Year 5 classes within the school were invited to participate. Special schools (for children whose additional needs cannot be met in a mainstream setting) were excluded because they were unlikely to be teaching the standard National Curriculum around which the intervention had been designed.

Of the 125 eligible primary schools, 44 expressed an immediate interest in being part of the study following a presentation at one of the DAPH meetings and four local learning community meetings to target schools in the most deprived wards. Of these 44 schools, 36 were eligible (the other 8 schools did not have a single Year 5 class) and 32 were purposely sampled to ensure that trial schools represented a range of number of Year 5 classes (1–3 Year 5 classes), locations (urban and rural) and deprivation (5–53% eligible for free school meals). In England and Wales children are eligible for free school meals (FSM) if their parents are on income support. Parents will receive income support if they have no or a low income (less than £16,190) or no more than £16,000 in savings. A partner’s income and savings will be taken into account. The percentage of children in a school eligible for free school meals are therefore used as a proxy measure of the general socio-economic status of children within that school. The National average of pupils eligible for free school meals at the time of recruiting schools into the trial (2012) was 19%. We aimed to have half of the schools in the trial above and below the national average.

For practical reasons, half the schools commenced the study in 2012 (Cohort 1, *n* = 658 children, 16 schools – 8 intervention and 8 control) and the other half in 2013 (Cohort 2, *n* = 666 children, 16 schools – 8 intervention and 8 control). The remaining four schools were asked if they were prepared to go on a ‘wait list’ in case one or more of the schools allocated to Cohort 2 dropped out during the waiting year; all four schools agreed to this.

Fourteen of the 32 schools recruited had ≥19% pupils eligible for free school meals. All schools were initially randomly allocated to intervention or control by computer-generated sequence stratified by (i) the proportion of children eligible for free school meals (<19%, ≥19%) and (ii) school size (one Year 5 class, >1 Year 5 class). Randomisation was performed by the UKCRC-registered Peninsula Clinical Trials Unit (PenCTU) immediately after all schools had been recruited (2012) but schools’ allocation (intervention or control) was not communicated to the schools, parents or researchers until ***after*** baseline measures had been taken for each respective cohort (2012 for Cohort 1 and 2013 for Cohort 2). Control schools continued with their usual curriculum, whilst the intervention schools received the HeLP programme.

### Outcome measures

All baseline measures were collected by the HeLP Coordinators and trained assessors prior to revealing schools’ allocated trial groups. All research staff underwent an enhanced Disclosure and Barring Service (DBS) check to ensure they were suitable to work with children prior to the start of the trial. Letters were sent home to parents prior to each set of data collection to remind them the measures were going to take place. Based on the learning from pilot work [22, 23] regarding what was most feasible and practical for schools and children, as well as being time efficient, measures were taken in the following order:

- Physical Activity (in one randomly selected class per school)
- Food Intake Questionnaire (FIQ)
- My Lifestyle Questionnaire (MLQ) (to assess potential mediating variables). This data will be presented in a separate paper.
- Anthropometric measures

Within each cohort, all measures were collected over an eight week time period. Physical activity measures and questionnaire data were collected in October. All anthropometric measures were collected over the course of one day in each school. If children were absent on the day of measurement, attempts to collect their data were taken for up to a further two weeks from the day of absence.

#### Anthropometric outcomes (baseline, 18 and 24 months)

Height was measured using a SECA stadiometer (Hamburg, Germany), recorded to an accuracy of 1 mm. Weight was measured using the Tanita Body Composition Analyser SC-330 (U.K. Ltd., Middlesex, U.K.). Weight was recorded to within 0.1 kg and children are asked to take off their shoes and socks and tights. Percent body fat was estimated from leg-to-leg bioelectric impedance analysis (Tanita Body Composition Analyser SC-330). Waist circumference was measured using a non-elastic flexible tape measure, 4 cm above the umbilicus. For each measure a detailed standard operating procedure was followed by the assessors, who underwent training prior to each measurement time point. The coefficients of variation to assess inter-rater reliability for height and waist circumference in the training session prior to the taking of baseline anthropometric measures were 0.1% and 1.2% respectively, suggesting a high level of precision.

In order to put children at ease and minimise any possible stigmatisation of overweight or sensitive children, these measurements formed part of a specially designed lesson which was based around measuring in general and how information can be presented. The HeLP Coordinator (HC) led the lesson which provided a good opportunity for them to learn the children’s names and for the children to become familiar with the HC. Each child, one at a time, left the classroom during the lesson to go to a private room and have their height, weight, waist circumference and percent body fat by bio-electrical impedance measured by two other trained researchers.

At each data collection time point, children had the option to decline one or more measurements if they so wished. For the anthropometric measures using the Tanita scales a printout is produced providing the child’s weight, BMI and % Body Fat. During measurements, the electronic reading was covered so that children were unable to read their results. This process had been developed during piloting to reduce any stigmatisation of overweight/obese and underweight children and any discussion about weight.

All anthropometric measures were taken by independent assessors blinded to group allocation at baseline, 18 and 24 months.

#### Behavioural outcomes (baseline and 18 months)

Physical activity form a subset of children (as described earlier) was objectively assessed using a triaxial accelerometer [24] worn continuously for 8 consecutive days on the wrist of the non-dominant arm. To assist with adherence, information packs were sent to parents a week prior to children being fitted with the accelerometers providing information on wearing the accelerometer and guidance to be distributed to sports coaches to prevent removal during sport. On the day of issue, HeLP Coordinators spoke to ten children at a time about how to comply with the procedures and answered any questions.

Food intake was assessed using the adapted version of the validated Food Intake Questionnaire (FIQ) [25]. A study to assess the FIQ’s validity and reliability [26] showed consistent responses on separate occasions for this age group over the 3-month reliability study period. Analysis of variance showed no differences in mean score for food groups between survey time points. Pearson correlations for mean scores estimated by separate FIQ ranged from 0.42 for fibre food group to 0.76 for negative marker food group; the majority of the correlations were above 0.5. These data suggest that the FIQ should be able to detect a change of ±10% in eating habits.

The FIQ asks children about the food and beverages they consumed the previous day and allows an estimation of the *number* of different types of healthy and unhealthy food and drink items consumed per day which equates to a portion. Children complete the FIQ twice in order to obtain a weekday (can be completed Tuesday to Friday) and weekend (can only be completed on a Monday) food intake. The HC led the two lessons required for the children to complete the questionnaires at each timepoint (baseline and 18 months). Children were arranged in literacy tables to ensure that assistance could be given as efficiently as possible. Another researcher, the class teacher and the teaching assistant (TA) also provided support.

All behavioural measures were taken blind to group allocation at baseline with partial blinding for physical actvity at 18 months (as it is an objective measure) whereas blinding for the FIQ at 18 months was not possible as it self-report during a lesson taken by the HeLP Coordinator.

### Data manipulations

#### Anthropometric outcomes

Standard Deviation Scores were derived for body mass index (BMI), based on the UK 1990 BMI reference curves for children [27]. Subsequently, each child was categorised as being underweight, healthy weight, overweight or obese, on the basis of his/her body mass index, using Cole’s 1990 BMI UK age and sex specific thresholds. Similarly, standard deviation scores were calculated for waist circumference, based on UK reference curves for waist circumference [28], with any child above the 90th centile for age and sex specific values derived from UK relevant centiles was defined as having central overweight/obesity, as suggested by the International Diabetes Federation [29], and for percentage body fat [30].

#### Behavioural outcomes

##### Food intake

Data from the FIQ were used to calculate the average number of different types of energy dense snacks (13 items), healthy snacks (10 items), negative food markers (25 items) and positive food markers (22 items) children consumed per day. All completed questionnaires were included in the analysis; 0.6% of the data were missing (i.e. children did not tick either yes or no). Details of the items in each category and how the questionnaire was scored with information on the management of missing values can be found at the project website [31].

##### Physical activity

Accelerometer’s were set to record at 85.7 Hz and data were downloaded using GeneActiv PC software version 1.4 and analysed using the GGIR software package for R [32]. Data were included for analysis if the accelerometer was worn for ≥10 h for ≥4 days (including ≥1 weekend day) [33]. Non-wear was determined using a published algorithm, details of which can be found elsewhere [34]. For participants who meet the minimum wear time criteria the data were again passed over in 60 min rolling windows with 45 min overlap to identify 15 min blocks of non-wear. The 15 min blocks were then imputed based on the average movement recorded in 15 min blocks at the same time of day for the whole monitoring [35]. For each participant a summary measure of acceleration based on the whole observation period was calculated by taking the vector magnitude of activity-related acceleration using the Euclidean Norm minus 1 g (ENMO; expressed in milligravity or m*g*) and converting negative numbers to zero [34]. Further time (minutes) spent in light, moderate and vigorous intensity physical activity, along with moderate to vigorous physical activity (MVPA) were obtained using published cut-points [36]. Time spent sedentary was estimated by summing minutes spent between 0 and 87.5 m*g* between the hours of 6 am and 10 pm to separate ‘sleep’ from sedentary time.

### Data analysis

Continuous measures were summarised by the mean, standard deviation and range, with frequencies and percentages reported for binary and categorical measures. Descriptive statistics of child level measures are presented for (a) all children, (b) by cohort, (c) by gender, as well as by the two stratification factors: (d) by number of Year 5 classes (one or more than one) and (e) by category of percentage of children eligible for free school meals (<19% vs ≥19%). No inferential statistical analysis was undertaken as there was no intention to formally compare measures within each of these presentation groups.

## Results

**Fig. 1:**
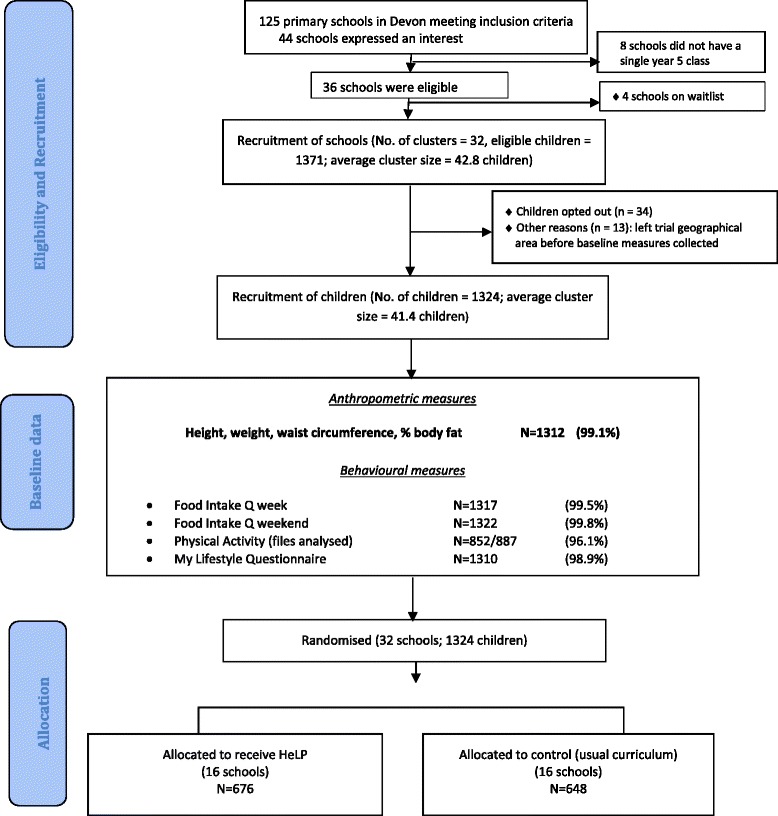
Recruitment of schools and children and completeness of baseline measures

We achieved 99% or more completeness of data for all anthropometric and behavioural measures at baseline. Figure 1 below shows the trial design, recruitment of schools and children and the numbers and percentages of data collected.

### Baseline characteristics of recruited schools and child demographics

Table 1 summarises the school characteristics and child demographic data. The mean percentage of children entitled to free school meals in the recruited schools was 20%, but ranged from 5.3% to 52.8%. As expected, given schools were balanced across cohorts, Cohort 1 and Cohort 2 were very similar in terms of school and child demographics.

**Table 1:** Demographics of recruited schools and children at baseline (numbers and percentages unless otherwise specified)

School characteristics		Cohort 1	Cohort 2	Total
Number of Participating Schools		16	16	32
Number of Year 5 Classes		24	27	51
Mean (sd) [range] Percentage of Free School Meals		20.3 (9.8) [6.1–37.8]	19.6 (13.5) [5.3–52.8]	20.0 (11.6) [5.3–52.8]
Child demographics				
Number of Participating Children		658	666	1324
Gender:	Girls	50.8 (334)	51.8 (345)	51.3 (679)
	Boys	49.2 (324)	48.2 (321)	48.7 (645)
Mean (sd) [range] of Age (years)^a^		9.8 (0.3) [9.2–10.8]	9.7 (0.3) [9.2–10.3]	9.8 (0.3) [9.2–10.8]

^a^Calculated as age at time of baseline anthropometric measures, only available for 1314 children

Table 2 shows the descriptive statistics of each anthropometric measure, in terms of both the raw data and the age/gender standardised measurements. The mean (standard deviation (sd)) BMI was 17.6 kg/m^2^ (2.9) and the mean BMI sds was 0.25 (1.16). Just less than three quarters of children were categorised as being of a healthy weight, with just over one quarter being overweight or obese.

**Table 2:** Baseline anthropometric measures (numbers and percentages unless otherwise specified) across recruited children and by stratification factors

	All children	Cohort 1	Cohort 2	Girls	Boys	One year 5 class^a^	More than one year 5 class^a^	FSM cat 1 (<19%)	FSM Cat2 (≥19%)
Mean (sd) [range] of BMI (kg/m^2^)	(*n* = 1312) 17.6 (2.9) [12.5–30.5]	(*n* = 655) 17.5 (2.9) [12.8–30.5]	(*n* = 657) 17.6 (2.8) [12.5–27.5]	(*n* = 672) 17.8 (3.0) [12.5–29.0]	(*n* = 640) 17.3 (2.6) [12.8–30.5]	(*n* = 554) 17.4 (2.8) [12.8–28.2]	(*n* = 758) 17.7 (2.9) [12.5–30.5]	(*n* = 816) 17.4 (2.7) [12.5–28.5]	(*n* = 496) 17.9 (3.1) [12.8–30.5]
Mean (sd) [range] of BMI sds	(*n* = 1312) 0.25 (1.16) [−2.88–3.36]	(*n* = 655) 0.21 (1.16) [−2.81–3.36]	(*n* = 657) 0.28 (1.15) [−2.88–3.18]	(*n* = 672) 0.26 (1.16) [−2.88–3.18]	(*n* = 640) 0.24 (1.16) [−2.81–3.36]	(*n* = 554) 0.16 (1.14) [−2.81–3.08]	(*n* = 758) 0.31 (1.17) [−2.88–3.36]	(*n* = 816) 0.19 (1.12) [−2.88–3.17]	(*n* = 496) 0.35 (1.20) [−2.81–3.36]
Underweight^a^	1.6 (21)	1.8 (12)	1.4 (9)	1.6 (11)	1.6 (10)	2.0 (11)	1.3 (10)	1.7 (14)	1.4 (7)
Healthy weight^a^	73.3 (962)	73.7 (483)	72.9 (479)	73.1 (491)	73.6 (471)	75.8 (420)	71.5 (542)	75.1 (613)	70.4 (349)
Overweight^a^	11.4 (150)	11.5 (75)	11.4 (75)	11.5 (77)	11.4 (73)	9.9 (55)	12.5 (95)	11.2 (91)	11.9 (59)
Obese^a^	13.6 (179)	13.0 (85)	14.3 (94)	13.8 (93)	13.4 (86)	12.3 (68)	14.6 (111)	12.0 (98)	16.3 (81)
Overweight or Obese^a^	25.1 (329)	24.4 (160)	25.7 (169)	25.3 (170)	24.8 (159)	22.2 (123)	27.2 (206)	23.2 (189)	28.2 (140)
Mean (sd) [range] of waist circumference (cm)	(*n* = 1309) 61.3 (7.5) [47.8–99.1]	(*n* = 655) 61.4 (7.8) [49.3–99.1]	(*n* = 654) 61.2 (7.2) [47.8–93.2]	(*n* = 672) 61.1 (7.5) [47.8–91.6]	(*n* = 637) 61.5 (7.5) [48.8–99.1]	(*n* = 552) 60.8 (7.1) [47.8–89.6]	(*n* = 757) 61.7 (7.7) [48.2–99.1]	(*n* = 816) 60.8 (6.8) [48.1–99.1]	(*n* = 493) 62.2 (8.4) [47.8–97.0]
Mean (sd) [range] of waist circumference sds	(*n* = 1309) 0.64 (1.13) [−2.53–3.82]	(*n* = 655) 0.64 (1.13) [−2.02–3.82]	(*n* = 654) 0.63 (1.13) [−2.53–3.77]	(*n* = 672) 0.69 (1.19) [−2.53–3.57]	(*n* = 637) 0.58 (1.07) [−2.21–3.82]	(*n* = 552) 0.56 (1.12) [−2.53–3.48]	(*n* = 757) 0.69 (1.14) [−2.30–3.82]	(*n* = 816) 0.57 (1.06) [−2.39–3.68]	(*n* = 493) 0.74 (1.23) [−2.53–3.82]
Mean (sd) [range] of % body fat	(*n* = 1309) 20.1 (7.1) [3.0–46.9]	(*n* = 654) 20.2 (7.1) [3.0–44.5]	(*n* = 655) 19.9 (7.1) [3.0–46.9]	(*n* = 671) 22.3 (6.9) [3.0–43.2]	(*n* = 638) 17.7 (6.4) [3.0–46.9]	(*n* = 554) 19.6 (6.8) [3.0–44.5]	(*n* = 755) 20.4 (7.2) [3.0–46.9]	(*n* = 814) 19.5 (6.9) [3.0–46.9]	(*n* = 495) 21.1 (7.2) [5.4–43.2]
Mean (sd) [range] of % body fat sds	(*n* = 1309) −0.62 (2.28) [−40.52–2.87]	(*n* = 654) −0.59 (2.53) [−40.52–2.87]	(*n* = 655) −0.64 (2.00) [−19.20–2.65]	(*n* = 671) −0.53 (2.15) [−19.20–2.87]	(*n* = 638) −0.71 (2.40) [−40.52–2.65]	(*n* = 554) −0.71 (2.00) [−18.81–2.64]	(*n* = 755) −0.55 (2.46) [−40.52–2.87]	(*n* = 814) −0.79 (2.51) [−40.52–2.65]	(*n* = 495) −0.34 (1.81) [−10.62–2.87]
Mean (sd) [range] of % body fat sds after exclusion of outliers^b^	(*n* = 1281) −0.42 (1.57) [−4.91–2.87]	(*n* = 642) −0.39 (1.57) [−4.91–2.87]	(*n* = 639) −0.46 (1.57) [−4.79–2.65]	(*n* = 656) −0.33 (1.59) [−4.91–2.87]	(*n* = 625) −0.52 (1.54) [−4.79–2.65]	(*n* = 543) −0.55 (1.56) [−4.76–2.64]	(*n* = 738) −0.33 (1.58) [−4.91–2.87]	(*n* = 794) −0.55 (1.57) [−4.86–2.65]	(*n* = 487) −0.22 (1.56) [−4.91–2.87]

^a^Categorisations based on the definitions for population monitoring of weight status in children by Public Health England [46]

^b^As defined by Cole, children with measurements greater than five standard deviations from the mean were omitted

Children from each cohort scored similarly on all anthropometric measures. There were few differences between boys and girls. Girls had higher mean waist circumference sds (0.69 (1.19) vs 0.58 (1.07)) and mean percentage body fat (22.3 (6.9) vs 17.7 (6.4)) than boys. There were some differences in anthropometric measures in terms of the category of number of Year 5 classes (one class vs more than one class): children in larger schools had a higher mean BMI sds (0.31 (1.17) vs 0.16 (1.14)) and waist circumference sds (0.69 (1.14) vs 0.56 (1.12)), and a greater proportion were classified as being overweight or obese (27.2% vs 22.2%), compared with children in schools with only one Year 5 class. This could be related to the fact that on average the larger schools had a slightly higher mean percentage of children eligible for free school meals (21.2% vs 18.9%). Similarly, children from schools with ≥19% FSM had a higher mean BMI sds (0.35 (1.20)) and waist circumference sds (0.74 (1.23)) compared with children from schools with <19% FSM (mean BMI sds of 0.19 (1.12); mean waist circumference sds of 0.57 (1.06)), and a higher percentage of children from schools with ≥19% FSM were categorised as overweight or obese (28.2% v 23.2%).

### Food intake data

Table 3 shows that food intake was similar across cohorts and that, on average, study children were consuming means of 4.15 (2.19) different energy dense snacks (EDS) and 3.23 (1.64) different healthy snacks (HS) per day, with slightly more varied EDS and less varied HS being eaten over the weekend. A similar ratio can be seen for consumption of healthy (Positive Food Markers) to unhealthy (Negative Food Markers) foods, where, on average, children consumed 5.85 (2.61) different healthy foods and 6.82 (3.38) different unhealthy foods per day, again with more varied unhealthy foods being consumed over the weekend. Slight differences were noted by gender with boys eating, on average, more varied energy dense snacks and unhealthy foods and less varied healthy snacks and healthy foods than girls. Children from schools with ≥19% FSM consumed more varied unhealthy snacks and foods and less varied healthy snacks and foods than children from schools with <19% FSM.

**Table 3:** Food intake questionnaire (numbers and percentages unless otherwise specified) across recruited children and by stratification factors

	All children	Cohort 1	Cohort 2	Girls	Boys	One year 5 class	More than one year 5 class	FSM cat 1 (<19%)	FSM cat2 (≥19%)
Mean (sd) [range] of Weekday Energy Dense Snacks (EDS)	(*n* = 1315) 4.01 (2.42) [0–13]	(*n* = 656) 4.13 (2.37) [0–13]	(*n* = 659) 3.88 (2.46) [0–13]	(*n* = 676) 3.82 (2.29) [0–13]	(*n* = 639) 4.20 (2.54) [0–13]	(*n* = 557) 4.06 (2.44) [0–13]	(*n* = 758) 3.97 (2.41) [0–13]	(*n* = 814) 3.78 (2.29) [0–13]	(*n* = 501) 4.36 (2.58) [0–13]
Mean (sd) [range] of Weekend Energy Dense Snacks (EDS)	(*n* = 1319) 4.49 (2.50) [0–13]	(*n* = 654) 4.53 (2.47) [0–12]	(*n* = 665) 4.45 (2.53) [0–13]	(*n* = 675) 4.32 (2.47) [0–13]	(*n* = 644) 4.67 (2.52) [0–13]	(*n* = 558) 4.39 (2.43) [0–13]	(*n* = 761) 4.56 (2.55) [0–13]	(*n* = 819) 4.39 (2.46) [0–13]	(*n* = 500) 4.65 (2.56) [0–13]
Mean (sd) [range] of Average Energy Dense Snacks (EDS)	(*n* = 1311) 4.15 (2.19) [0–13]	(*n* = 653) 4.25 (2.14) [0–12.71]	(*n* = 658) 4.04 (2.23) [0–13]	(*n* = 672) 3.97 (2.08) [0–13]	(*n* = 639) 4.34 (2.28) [0–13]	(*n* = 554) 4.16 (2.16) [0–13]	(*n* = 757) 4.14 (2.21) [0–13]	(*n* = 813) 3.96 (2.10) [0–12.71]	(*n* = 498) 4.46 (2.29) [0–13]
Mean (sd) [range] of Weekday Healthy Snacks (HS)	(*n* = 1310) 3.27 (1.77) [0–10]	(*n* = 653) 3.34 (1.77) [0–10]	(*n* = 657) 3.19 (1.77) [0–10]	(*n* = 673) 3.33 (1.75) [0–9]	(*n* = 637) 3.20 (1.80) [0–10]	(*n* = 555) 3.46 (1.80) [0–10]	(*n* = 755) 3.13 (1.74) [0–10]	(*n* = 813) 3.40 (1.74) [0–10]	(*n* = 497) 3.06 (1.80) [0–9]
Mean (sd) [range] of Weekend Healthy Snacks (HS)	(*n* = 1306) 3.09 (1.89) [0–10]	(*n* = 649) 3.02 (1.85) [0–10]	(*n* = 657) 3.16 (1.91) [0–10]	(*n* = 674) 3.15 (1.90) [0–10]	(*n* = 632) 3.03 (1.87) [0–10]	(*n* = 550) 3.10 (1.92) [0–10]	(*n* = 756) 3.04 (1.87) [0–10]	(*n* = 810) 3.32 (1.87) [0–10]	(*n* = 496) 2.72 (1.85) [0–10]
Mean (sd) [range] of Average Healthy Snacks (HS)	(*n* = 1294) 3.23 (1.64) [0–9.14]	(*n* = 645) 3.27 (1.63) [0–9.14]	(*n* = 649) 3.19 (1.64) [0–8.86]	(*n* = 668) 3.29 (1.62) [0–8.43]	(*n* = 626) 3.16 (1.65) [0–9.14]	(*n* = 554) 3.40 (1.64) [0–9.14]	(*n* = 749) 3.10 (1.62) [0–8.86]	(*n* = 804) 3.38 (1.62) [0–9.14]	(*n* = 490) 2.98 (1.64) [0–8.43]
Mean (sd) [range] of Weekday Positive Markers (PM)	(*n* = 1315) 5.88 (2.83) [0–20]	(*n* = 655) 5.93 (2.79) [0–16]	(*n* = 660) 5.83 (2.88) [0–20]	(*n* = 675) 5.94 (2.70) [0–20]	(*n* = 640) 4.82 (2.97) [0–18]	(*n* = 556) 6.17 (2.93) [0–20]	(*n* = 759) 5.66 (2.74) [0–18]	(*n* = 816) 5.98 (2.69) [0–18]	(*n* = 499) 5.72 (3.05) [0–20]
Mean (sd) [range] of Weekend Positive Markers (PM)	(*n* = 1314) 5.78 (2.97) [0–20]	(*n* = 653) 5.68 (2.85) [0–17.81]	(*n* = 661) 5.88 (3.08) [0–20]	(*n* = 676) 5.81 (2.88) [0–18.70]	(*n* = 638) 5.75 (3.07) [0–20]	(*n* = 556) 6.17 (2.93) [0–20]	(*n* = 759) 5.66 (2.74) [0–18]	(*n* = 816) 6.05 (2.88) [0–20]	(*n* = 498) 5.34 (3.07) [0–18.70]
Mean (sd) [range] of Average Positive Markers (PM)	(*n* = 1306) 5.85 (2.61) [0–19.56]	(*n* = 651) 5.86 (2.56) [0–16.14]	(*n* = 655) 5.84 (2.67) [0–19.56]	(*n* = 672) 5.91 (2.50) [0–19.56]	(*n* = 634) 5.79 (2.73) [0–16.57]	(*n* = 550) 6.11 (2.67) [0.29–19.56]	(*n* = 756) 5.66 (2.55) [0–16.57]	(*n* = 812) 5.99 (2.50) [0–16.57]	(*n* = 494) 5.62 (2.78) [0–19.56]
Mean (sd) [range] of Weekday Negative Markers (NM)	(*n* = 1316) 6.58 (3.74) [0–24]	(*n* = 656) 6.79 (3.70) [0–24]	(*n* = 660) 6.37 (3.76) [0–23]	(*n* = 676) 6.25 (3.45) [0–23]	(*n* = 640) 6.93 (3.99) [0–24]	(*n* = 557) 6.71 (3.82) [0–23]	(*n* = 759) 6.48 (3.67) [0–24]	(*n* = 815) 6.11 (3.44) [0–24]	(*n* = 501) 7.34 (4.06) [1–23]
Mean (sd) [range] of Weekend Negative Markers (NM)	(*n* = 1320) 7.41 (3.78) [0–23]	(*n* = 655) 7.33 (3.70) [0–22]	(*n* = 665) 7.49 (3.87) [0–23]	(*n* = 676) 7.12 (3.60) [0–23]	(*n* = 644) 7.71 (3.95) [0–23]	(*n* = 558) 7.27 (3.75) [0–23]	(*n* = 762) 7.52 (3.81) [0–23]	(*n* = 820) 7.28 (3.74) [0–23]	(*n* = 500) 7.63 (3.85) [0–23]
Mean (sd) [range] of Average Negative Markers (NM)	(*n* = 1313) 6.82 (3.38) [0.57–23.43]	(*n* = 654) 6.95 (3.32) [0.57–23.43]	(*n* = 659) 6.68 (3.43) [0.60–22.71]	(*n* = 673) 6.50 (3.12) [0.57–22.71]	(*n* = 640) 7.15 (3.60) [0.60–23.43]	(*n* = 554) 6.87 (3.39) [0.57–22.71]	(*n* = 759) 6.78 (3.37) [0.57–23.43]	(*n* = 815) 6.44 (3.18) [0.57–23.43]	(*n* = 498) 7.44 (3.58) [1.00–22.71]

### Physical activity

Across the cohorts, 886 children were randomised to receive a GeneActiv watch at baseline, 851 (96%) files were analysed and 830 of these files (97.5%) files met the inclusion criteria of 4 days (including one weekend day of at least 10 h wear time). Compliance was also excellent for 7 days of 10 h wear time (94.8%), allowing us to accurately estimate the percentage of children achieving the Department of Health’s physical activity guidelines [37]. Table 4 shows that mean levels of physical activity per day for all intensities were similar across cohorts and by FSM category. On average, children spent 53.6 min in MVPA, although this ranged from 11.9 to 124.8 min. Boys spent more time in all physical activity intensities than girls, except the light category, with the greatest difference being in mean MVPA (59.9 (17.3) vs 48.2 (13.7) mins). There was also a slight gender difference in the average magnitude of acceleration (45.4 (8.8) m*g* for girls and 53.6 (11.8) m*g* for boys). On average, based on these timings, children spend 13.0 (0.6) hours a day under the light intensity threshold. Girls were slightly more inactive than boys but this difference was minimal.

**Table 4:** Baseline physical activity measures (numbers and percentages unless otherwise specified) across sub-group of recruited children and by stratification factors

	All children	Cohort 1	Cohort 2	Girls	Boys	One year 5 class	More than one year 5 class	FSM cat 1 (<19%)	FSM Cat2 (≥19%)
Mean (sd) [range] Physical Activity - Sedentary (6 am to 10 pm) (minutes)	(*n* = 830) 779.3 (35.1) [671.0–893.9]	(*n* = 421) 781.3 (34.8) [671.0–859.0]	(*n* = 409) 777.2 (35.3) [680.6–893.9]	(*n* = 434) 784.2 (33.0) [692.8–893.9]	(*n* = 396) 773.9 (36.6) [671.0–865.6]	(*n* = 492) 780.3 (35.5) [671.0–865.6]	(*n* = 338) 777.8 (34.5) [683.4–893.9]	(*n* = 471) 778.1 (34.7) [680.6–893.9]	(*n* = 359) 780.8 (35.6) [671.0–865.6]
Mean (sd) [range] of Physical Activity - Light (minutes)	(*n* = 830) 130.3 (24.4) [58.0–211.4]	(*n* = 421) 128.9 (23.9) [75.6–206.6]	(*n* = 409) 131.6 (24.9) [58.0–211.4]	(*n* = 434) 130.9 (23.8) [58.0–211.4]	(*n* = 396) 129.6 (25.1) [69.5–199.8]	(*n* = 492) 129.0 (24.0) [69.5–211.4]	(*n* = 338) 132.1 (24.9) [58.0–206.6]	(*n* = 471) 130.7 (24.1) [58.0–211.4]	(*n* = 359) 129.7 (24.9) [74.0–206.6]
Mean (sd) [range] of Physical Activity - Moderate (minutes)	(*n* = 830) 40.2 (11.7) [9.9–85.9]	(*n* = 421) 39.5 (11.7) [13.7–85.9]	(*n* = 409) 41.0 (11.7) [9.9–81.4]	(*n* = 434) 37.4 (10.4) [9.9–72.2]	(*n* = 396) 43.3 (12.4) [15.0–85.9]	(*n* = 492) 40.3 (12.1) [15.0–80.3]	(*n* = 338) 40.2 (11.3) [9.9–85.9]	(*n* = 471) 40.5 (11.8) [9.9–85.9]	(*n* = 359) 39.9 (11.7) [15.0–80.3]
Mean (sd) [range] of Physical Activity - Vigorous (minutes)	(*n* = 830) 13.4 (6.2) [2.0–51.1]	(*n* = 421) 13.5 (6.6) [2.1–51.1]	(*n* = 409) 13.3 (5.7) [2.0–37.6]	(*n* = 434) 10.8 (4.4) [2.0–34.1]	(*n* = 396) 16.3 (6.5) [2.8–51.1]	(*n* = 492) 13.5 (6.3) [2.4–51.1]	(*n* = 338) 13.3 (6.0) [2.0–38.4]	(*n* = 471) 13.6 (6.0) [2.0–38.4]	(*n* = 359) 13.2 (6.3) [2.1–51.1]
Mean (sd) [range] of Physical Activity - MVPA (minutes)	(*n* = 830) 53.6 (16.5) [11.9–124.8]	(*n* = 421) 53.0 (17.1) [20.0–124.8]	(*n* = 409) 54.3 (15.9) [11.9–106.3]	(*n* = 434) 48.2 (13.7) [11.9–92.1]	(*n* = 396) 59.9 (17.3) [20.0–124.8]	(*n* = 492) 53.7 (17.0) [20.0–124.8]	(*n* = 338) 53.5 (15.9) [11.9–124.3]	(*n* = 471) 54.1 (16.4) [11.9–124.3]	(*n* = 359) 53.0 (16.6) [20.9–124.8]
Mean (sd) [range] of m*g* value^a^	(*n* = 830) 49.3 (11.1) [18.3–105.4]	(*n* = 421) 49.0 (11.5) [27.4–105.4]	(*n* = 409) 49.6 (10.7) [18.3–85.8]	(*n* = 434) 45.4 (8.8) [18.3–76.7]	(*n* = 396) 53.6 (11.8) [24.8–105.4]	(*n* = 492) 49.2 (11.4) [24.8–105.4]	(*n* = 338) 49.4 (10.8) [18.3–91.8]	(*n* = 471) 49.7 (11.0) [18.3–91.8]	(*n* = 359) 48.8 (11.3) [24.8–105.4]

^a^GeneActiv measures the acceleration of movement at the wrist and provides the output as raw acceleration (milli-*g*, i.e. multiples of 0.00981 m/s^2^). With previous accelerometry monitors, raw acceleration data was converted into counts through a manufacturer-specific algorithm that wasn’t disclosed, preventing comparison between studies. The average acceleration for each child provides a measure of acceleration over the measurement period

## Discussion

The percentage of children eligible for free school meals in a school in England can be used a proxy for the socio-economic status of children within that school. In England the average is 19% meaning that schools with percentages above this are considered to contain more children from deprived backgrounds than those with less than 19%. A strength of the HeLP trial design was the aim that half of all schools included in the study would have ≥19% FSM so that clusters were a representative of national primary schools, for socio-economic status. Childhood obesity is strongly correlated socio-economic status [1] thus interventions should always be mindful of the need to engage children and their families across the social spectrum and evaluations need to include children from all SES groups to ensure that interventions do not increase health inequalities. The percentage of children eligible for free school meals in the 32 participating schools ranged from 5.3% and 52.8%, suggesting that we have conducted the research on a nationally representative sample of schools in terms of SES/FSM.

### Anthropometric data

Table 2 shows that children from each cohort scored similarly on all anthropometric measures. In the different weight categories our results are slightly lower in terms of proportion of children who are obese or overweight, than the National and local data from the NCMP 2014/15 [1]. All data show that a greater proportion of children are obese than are overweight (NCMP (England) – 14.1% overweight and 19.1% obese; NCMP (Devon) - 13.5% overweight and 14.2% obese; HeLP baseline data - 11.4% overweight and 13.6% obese). The slightly lower figures for the HeLP baseline data are unsurprising considering our data are from children who are a year younger than the Year 6 NCMP data. The Devon NCMP data shows that a quarter of children are categorised as overweight and obese when they enter primary school which increases to 27.7% by the time they leave primary school (nationally, 21.9% and 33.2% respectively). In line with local and national data [38] differences in the overweight and obese categories were seen by gender and percentage of children eligible for free school meals (FSM) (<19%; ≥19%). A slightly higher percentage of girls were overweight/obese than boys (25.3% v 24.8%) and a higher percentage of children from schools with ≥19% FSM were categorised as overweight or obese (28.2% v 23.2%). Furthermore, these schools also had less children categorised in the healthy weight category (70.4% v 75.1%). The percentage of children categorised as underweight (1.6%) was similar to the NCMP results for Year 6 children (1.3%).

The average BMI sds score for all children was 0.25, meaning that compared to Coles, 1998 reference curves [27], children are over 0.25 standard deviation scores above the average BMI of the referent population in 1990. There was a marked difference in BMI sds score for the FSM categories with children attending schools with ≥19% FSM having a 0.35 sds score compared to 0.19 for children attending schools with <19% FSM.

The average waist circumference was similar for girls and boys (61.1 cm v 61.5 cm respectively). The average waist circumference sds score was 0.64 (with girls and children from schools with <19% pupil eligible for FSM showing a larger increase against the referent population) suggesting that children’s waistlines have increased more markedly than BMI in the last 25 years. A cross sectional examination of waist circumference in 11–16 year olds in a study by McCarthy et al. in 2003 [39], found that waist circumference had increased much faster than BMI over a 10–20 year period with the authors concluding that BMI might systematically underestimate the prevalence of obesity in children as it measures the sum of both the fat and fat free mass. Waist circumference would therefore appear to be a useful addition to BMI as outcome measure.

The average percentage body fat was 20.1% with girls having a higher proportion of %body fat compared to boys (22.3% v 17.7%). The average percentage body fat sds score for children across all categories was negative suggesting that compared to the reference curves of 2006, children’s percentage fat mass has decreased, contradicting the standardised BMI and WC findings. However, the 2006 references curves carried out analysis on only 1985 children across a wide age range (5 to 18.5 years), and the number of children in each age category is not reported. In addition to the limitations of the % body fat reference population, there are also limitations associated with using bioelectrical impedance analysis to measure body fatness among young people [40], which is why it is rarely used as a *primary* outcome measure in trials of public health interventions. Few studies have assessed all three measures of adiposity, suggesting that studies which monitor all three anthropometric outcomes are needed to better understand the relationship between these measurements.

### Food intake data

Food intake data show that children are consuming approximately 1 healthy snack to 1.3 energy dense snacks per day, with an increase in the number of different types of energy dense snacks consumed and decrease in the number of different types of healthy snacks consumed over the weekend. A similar ratio and week to weekend pattern was seen for healthy and unhealthy foods. Boys consumed slightly more varied unhealthy foods and slightly less varied healthy foods than girls. This pattern was repeated for children in FSM category 2 compared to FSM category 1.

It is difficult to compare our snacking data and positive and negative food markers data directly with other studies as there is not a standardised questionnaire. The FIQ was adapted for the HeLP trial to capture the food intake data relevant to the intervention. The HeLP data are in agreement with findings from the National Diet and Nutrition Survey (NDNS) [41]. Mean intake of non-milk extrinsic sugars (NMES) in the NDNS was found to exceed the recommendation of no more than 11% of food energy. In children aged 4 to 10 years and 11 to 18 years mean intake of NMES provided 14.7% and 15.6% of food energy respectively and came mainly from soft drinks and fruit juice. Soft drinks alone provided 30% of the NMES intake in the 11 to 18 years age group. Cereals, cakes and biscuits were the other major contributors. In addition, fruit and vegetable consumption in the NDNS (foods included in the positive food marker category in our study), showed that 9–12 year olds were least likely to eat the recommended five or more portions a day. Girls and children from highest income quintile, on average, consumed more portions than boys and children from the lowest income quintile and this is reflected in the baseline HeLP data whereby boys and children from FSM category 2 consumed slightly more unhealthy foods and slightly less healthy foods than girls and children from FSM category 1.

### Physical activity

Although differences were observed by both gender and free school meal category for food intake, only gender differences were observed for physical activity. Data from other studies using the GeneActiv device with children of a similar age allow comparison between mean levels of raw acceleration (m*g*) per day. Comparisons of time spent at different intensities is not possible due to differences in thresholds used. Our data of 45.4 m*g* for girls and 53.6 m*g* for boys is comparable to Da silva and Colleagues [42] who reported mean acceleration in children two years younger (55.1 m*g* in girls and 64.6 m*g* in boys) [42] and Fairclough et al., (2016) who reported mean acceleration values of 47.31 m*g*, in children of the same age [43].

A strength of the present study is the high compliance with the minimum wear time criteria (97.4% at ≥10 h wear for ≥3 weekdays and one weekend day) for inclusion in the analysis. It compares favourably with other studies using waist worn accelerometers with children in the same age range. For example, the ALSPAC study had 84.5% compliance using ≥3 days of 10 h wear time [6], the Millennium cohort study had 67% compliance using ≥2 days of 10 h wear time [44] and the Active for Life Year 5 trial had 60% compliance using ≥3 days of 8 h wear time [15].

As 94.8% (807/851) of children wore the accelerometer for 7 days with at least 10 h of continuous wear time, we were able to accurately estimate whether children were meeting the Department of Health’s recommendation that children spend ≥60 min each day doing moderate to vigorous intensity physical activity [37]. Most studies are unable to report this data as very few children comply at this threshold, so tend to calculate the percentage of children achieving an average of 60 min of MVPA per day. The most recent population data for English children comes from the 2008 Health Survey for England who reported that 33% of boys and 21% of girls achieved the recommended daily level of physical activity, despite this being based on children averaging at least 60 min on each day [45]. In addition, the analysis included only a very small subset of children aged 4 to 15 using a waist worn accelerometer, rather than the GeneActiv monitor used in the present study. Our data, which is from the largest representative group of 9–10 year old children to date with 7 days of data, reveal that only 5.5% of boys and 1.2% of girls achieved the recommended minimum of 60 min of MVPA on all days of the week, considerably lower than the proportion averaging at least 60 min of MVPA per day. This distinction is clearly important especially as many studies with low compliance only report the proportion of children averaging 60 min per day of MVPA, potentially leading to an overestimation of the proportion of children meeting the DoH recommendations. More studies which are able to obtain complete 7 day objectively measured physical activity data are required to confirm the findings from this study in relation to how much physical activity children are doing as opposed to relying on estimations based on 1–3 days of data. This has a major implication for public health guidance regarding children’s activity recommendations. If only circa 3% of children are reaching the DoH guidelines in any one day, this would call into question whether the recommended 60 min a day is a realistic public health target.

## Conclusion

We have anthropometric, diet and physical activity data on 99.5% of children participating in the Healthy Lifestyles Programme trial in the SW England. Furthermore, we have recruited children from schools of varying socioeconomic status with 14 of the 32 schools have ≥19% pupils eligible for free school meals (the national average at the start of the trial). The baseline data suggests that our trial children are representative of children in the South West of England and the UK in terms of their weight status, diet, physical activity and sedentary behaviours. In summary, one quarter of children are overweight/obese, 5% lower than the national figures using the NCMP data. This, however, is to be expected as our data is taken from children who are one year younger. Our data show that the percentage of children categorised as overweight/obese was greater for girls than it was boys, however this difference was small (25.3% v 24.8%). We could hypothesise that this may be due to differences observed in physical activity, particularly for the moderate to vigorous intensity, which was greater for boys by an average of ten minutes a day. In terms of socio- economic status, the percentage of children categorised as overweight/obese was greater in children in more deprived schools. We could hypothesise here that this may be due to the observed differences in food intake, with children from schools in FSM category 2 consuming more varied unhealthy and less varied healthy snacks and foods than children from schools in FSM category 1 by approximately one type per day.

It is difficult to directly compare our diet data with the National Diet and Nutrition Survey (NDNS) as we used an adapted version of the Food Intake Questionnaire to specifically assess intake related to the key behavioural messages in the HeLP intervention (namely the number of different types of healthy to unhealthy snacks and foods consumed per day). Our data do however, support data from the NDNS, in that children are consuming too many non-milk extrinsic sugars (e.g. sugary drinks, cakes, cereals biscuits, chocolate). The physical activity data is the most comprehensive of any available for children in this age group as we achieved 97.5% compliance at our specified minimum threshold of 4 days (including one weekend day) of at least 10 h wear time. In addition, we were able to include 807 (94.8%) children in the analysis to ascertain the percentage of achieving the Departments of Health’s recommendation of at least 60 min of MVPA each day, a sample size that has not been achieved in any national studies to date. The HSE, 2008 based their calculations on children averaging at least 60 min per day across the week, which is not the same as ascertaining whether children were actually doing 60 min of MVPA each day of the week. For this calculation, complete data for all seven days is required meaning we able to carry out this calculation. The resultant percentages were far lower than those of the HSE 2008 (5.5% v 33% of boys and 1.2% v 21% of girls) suggesting that, when only small sample sizes are available, due to low wear time compliance, overestimates of the prevalence of physical activity at recommended levels in children is likely.

## Abbreviations

AfLY5: Active for life year 5
ALSPAC: Avon longitudinal study of parents and children
BCTs: Behaviour change techniques
BMI: Body mass index
DAPH: Devon Association of Primary Head teachers
DBS: Disclosure and Barring Service
EDS: Energy dense snack
ENMO: Euclidean Norm minus one
FIQ: Food intake questionnaire
FSM category 1: Schools with <19% of pupils eligible for free school meals
FSM category 2: Schools with ≥19% of pupils eligible for free school meals
FSM: Free school meals
HC: HeLP Coordinator
HeLP: Healthy Lifestyles Programme
HPS: Health promoting schools
HS: Healthy snack
IQR: Inter quartile range
LMS method: A statistical method to smooth BMI centile reference curves
m*g*: Milligravity
MLQ: My lifestyle questionnaire
MRC: Medical research council
MVPA: Moderate to vigorous physical activity
NCMP: National Child Measurement Programme
NDNS: National Diet and Nutrition Survey
NM: Negative food marker
NMES: Non milk extrinsic Ssugars
Pen CTU: Peninsula Clinical Trials Unit
PM: Positive food marker
Sd: Standard deviation
sds: Standard deviation scores
WHO: World Health Organisation

## References

[1] National Child Measurement Programme. England, 2014/15 school year. [http://www.hscic.gov.uk/catalogue/PUB16070/nati-chil-meas-prog-eng-2013-2014-rep.pdf]. Accessed 11 Jan 2016.

[2] Global strategy on diet, physical activity and health: childhood overweight and obesity. [http://www.who.int/dietphysicalactivity/childhood/en/]. Accessed 18 Dec 2014.

[3] Reilly JJ, Ness AR, Sherriff A (2007). Epidemiological and physiological approaches to understanding the etiology of pediatric obesity: finding the needle in the haystack. Pediatr Res.

[4] Wareham N (2007). Physical activity and obesity prevention. Obes Rev.

[5] Marshall SJ, Biddle SJ, Gorely T, Cameron N, Murdey I (2004). Relationships between media use, body fatness and physical activity in children and youth: a meta-analysis. Int J Obes Relat Metab Disord.

[6] Ness AR, Leary SD, Mattocks C, Blair SN, Reilly JJ, Wells J, Ingle S, Tilling K, Smith GD, Riddoch C (2007). Objectively measured physical activity and fat mass in a large cohort of children. PLoS Med.

[7] Mattocks C, Ness A, Deere K, Tilling K, Leary S, Blair SN, Riddoch C (2008). Early life determinants of physical activity in 11 to 12 year olds: cohort study. BMJ.

[8] Ludwig DS, Peterson KE, Gortmaker SL (2001). Relation between consumption of sugar-sweetened drinks and childhood obesity: a prospective, observational analysis. Lancet.

[9] Gazzaniga JM, Burns TL (1993). Relationship between diet composition and body fatness, with adjustment for resting energy expenditure and physical activity, in preadolescent children. Am J Clin Nutr.

[10] Malik VS, Schulze MB, Hu FB (2006). Intake of sugar-sweetened beverages and weight gain: a systematic review. Am J Clin Nutr.

[11] Gonzalez-Suarez C, Worley A, Grimmer-Somers K, Dones V (2009). School-based interventions on childhood obesity: a meta-analysis. Am J Prev Med.

[12] Langford R, Bonell C, Jones H, Pouliou T, Murphy S, Waters E, Komro K, Gibbs L, Magnus D, Campbell R (2015). The World Health Organization's Health Promoting Schools framework: a Cochrane systematic review and meta-analysis. BMC Public Health.

[13] Wang Y, Cai L, Wu Y, Wilson RF, Weston C, Fawole O, Bleich SN, Cheskin LJ, Showell NN, Lau BD (2015). What childhood obesity prevention programmes work? A systematic review and meta-analysis. Obes Rev.

[14] Khambalia AZ, Dickinson S, Hardy LL, Gill T, Baur LA (2012). A synthesis of existing systematic reviews and meta-analyses of school-based behavioural interventions for controlling and preventing obesity. Obes Rev.

[15] Kipping RR, Howe LD, Jago R, Campbell R, Wells S, Chittleborough CR, Mytton J, Noble SM, Peters TJ, Lawlor DA (2014). Effect of intervention aimed at increasing physical activity, reducing sedentary behaviour, and increasing fruit and vegetable consumption in children: active for Life Year 5 (AFLY5) school based cluster randomised controlled trial. BMJ.

[16] Jago R, Rawlins E, Kipping RR, Wells S, Chittleborough C, Peters TJ, Mytton J, Lawlor DA, Campbell R (2015). Lessons learned from the AFLY5 RCT process evaluation: implications for the design of physical activity and nutrition interventions in schools. BMC Public Health.

[17] Craig P, Dieppe P, Macintyre S, Michie S, Nazareth I, Petticrew M (2008). Developing and evaluating complex interventions: the new Medical Research Council guidance. Br Med J.

[18] Bartholomew LK, Markham CM, Ruiter R, Fernández ME, Kok G, Parcel GS (2016). Planning Health Promotion Programmes An Intervention mapping Approach.

[19] Lloyd JJ, Logan S, Greaves CJ, Wyatt KM (2011). Evidence, theory and context - using intervention mapping to develop a school-based intervention to prevent obesity in children. Int J Behav Nutr Phys Act.

[20] Lloyd J, Wyatt K (2015). The Healthy Lifestyles Programme (HeLP) - An Overview of and Recommendations Arising from the Conceptualisation and Development of an Innovative Approach to Promoting Healthy Lifestyles for Children and Their Families. Int J Environ Res Public Health.

[21] Wyatt KM, Lloyd JJ, Abraham C, Creanor S, Dean S, Densham E, Daurge W, Green C, Hillsdon M, Pearson V (2013). The Healthy Lifestyles Programme (HeLP), a novel school-based intervention to prevent obesity in school children: study protocol for a randomised controlled trial. Trials.

[22] Lloyd JJ, Wyatt KM (2014). Qualitative findings from an exploratory trial of the Healthy Lifestyles Programme (HeLP) and their implications for the process evaluation in the definitive trial. BMC Public Health.

[23] Wyatt KM, Lloyd JJ, Creanor S, Logan S. The development, feasibility and acceptability of a school-based obesity prevention programme: results from three phases of piloting. BMJ Open. 2011;1(1). Available from: http://bmjopen.bmj.com/content/early/2011/05/23/bmjopen-2010-000026.abstract. 10.1136/bmjopen-2010-000026 PMC3191390 22021732

[24] GENEActiv. [www.geneactiv.org/]. Accessed 1 Mar 2016.

[25] Johnson B, Hackett AF. Eating habits of 11–14-year-old schoolchildren living in less affluent areas of Liverpool, UK. J Human Nutr Dietetics 1997;10(2):135-144.

[26] Johnson B, Hackett A, Roundfield M, Coufopoulos A (2001). An investigation of the validity and reliability of a food intake questionnaire. J Hum Nutr Diet.

[27] Cole TJ, Freeman JV, Preece MA (1998). British 1990 growth reference centiles for weight, height, body mass index and head circumference fitted by maximum penalized likelihood. StatMed.

[28] McCarthy HD, Jarrett KV, Crawley HF (2001). The development of waist circumference percentiles in British children aged 5.0-16.9 y. Eur J Clin Nutr.

[29] Zimmet P, Alberti G, Kaufman F, Tajima N, Silink M, Arslanian S, Wong G, Bennett P, Shaw J, Caprio S (2007). The metabolic syndrome in children and adolescents. Lancet.

[30] McCarthy HD, Cole TJ, Fry T, Jebb SA, Prentice AM (2006). Body fat reference curves for children. Int J Obes.

[31] National Institute for Health Research Project PHR - 10/3010/01. [http://www.nets.nihr.ac.uk/projects/phr/10301001]. Accessed 01 Mar 2016.

[32] van Hees VT, Zhao JH, Sabia S. Package ‘GGIR’: Raw Accelerometer Data Analysis. https://cran.r-project.org/web/packages/GGIR/index.html.

[33] Trost SG, McIver KL, Pate RR (2005). Conducting accelerometer-based activity assessments in field-based research. Med Sci Sports Exerc.

[34] van Hees VT, Gorzelniak L, Dean Leon EC, Eder M, Pias M, Taherian S, Ekelund U, Renstrom F, Franks PW, Horsch A (2013). Separating movement and gravity components in an acceleration signal and implications for the assessment of human daily physical activity. PLoS One.

[35] Sabia S, van Hees VT, Shipley MJ, Trenell MI, Hagger-Johnson G, Elbaz A, Kivimaki M, Singh-Manoux A (2014). Association Between Questionnaire- and Accelerometer-Assessed Physical Activity: The Role of Sociodemographic Factors. Am J Epidemiol.

[36] Phillips LRS, Parfitt G, Rowlands AV (2013). Calibration of the GENEA accelerometer for assessment of physical activity intensity in children. J Sci Med Sport.

[37] Bull FC, the Expert Working Groups. Physical Activity Guidelines in the U.K.: Review and Recommendations. School of Sport Exercise and Health Sciences Loughborough University. 2010. https://www.gov.uk/government/uploads/system/uploads/attachment_data/file/213743/dh_128255.pdf. Accessed 16 Feb 2016.

[38] Statistics on Obesity, Physical Activity and Diet: England 2015. [http://www.hscic.gov.uk/catalogue/PUB16988/obes-phys-acti-diet-eng-2015.pdf]. Accessed 01 Mar 2016.

[39] McCarthy HD, Ellis SM, Cole TJ (2003). Central overweight and obesity in British youth aged 11-16 years: cross sectional surveys of waist circumference. BMJ.

[40] Talma H, Chinapaw MJ, Bakker B, HiraSing RA, Terwee CB, Altenburg TM (2013). Bioelectrical impedance analysis to estimate body composition in children and adolescents: a systematic review and evidence appraisal of validity, responsiveness, reliability and measurement error. Obes Rev.

[41] Public Health England. National Diet Nutrition Survey: results from years 1, 2, 3 and 4 (combined) of the rolling programme (2008/09–2011/12). 2014. http://www.mrc-hnr.cam.ac.uk/research/nutrition-surveys-and-studies/national-diet-and-nutrition-survey/. Accessed 25 Jan 2016.

[42] da Silva IC, van Hees VT, Ramires VV, Knuth AG, Bielemann RM, Ekelund U, Brage S, Hallal PC (2014). Physical activity levels in three Brazilian birth cohorts as assessed with raw triaxial wrist accelerometry. Int J Epidemiol.

[43] Fairclough SJ, Noonan R, Rowlands AV, Vanh V, Knowles Z, Boddy LM (2016). Wear Compliance and Activity in Children Wearing Wrist- and Hip-Mounted Accelerometers. Med Sci Sports Exerc.

[44] Griffiths LJ, Cortina-Borja M, Sera F, Pouliou T, Geraci M, Rich C, Cole TJ, Law C, Joshi H, Ness AR, et al. How active are our children? Findings from the Millennium Cohort Study. BMJ Open. 2013;3(8):e002893. doi:10.1136/bmjopen-2013-002893. 10.1136/bmjopen-2013-002893 PMC3752053 23965931

[45] Esliger D, Hall J. Accelerometry in children. In: Health Survey for England 2008: Volume 1 Physical activity and fitness. vol. 1: The NHS Information Centre for health and social care; 2009: 159–180.

[46] Measuring and interpreting BMI in Children. [www.noo.org.uk/NOO_about_obesity/measurement/children]. Accessed 10 Mar 2016.

